# On GVC and innovation: the moderating role of policy

**DOI:** 10.1007/s40812-022-00255-9

**Published:** 2023-01-03

**Authors:** Yasmine Eissa, Chahir Zaki

**Affiliations:** 1 Faculty of Economics and Political Science, Cairo University, Cairo, Egypt; 2 Economic Research Forum, Cairo, Egypt

**Keywords:** Global value chains, Innovation, R&D, Technological change, F14, O31

## Abstract

This paper empirically investigates the association between global value chains (GVC) participation and countries’ innovation performance. Highlighting the learning effect of foreign knowledge embedded in imported intermediate goods counters the argument that GVC participation is biased towards developed countries with skilled labor abundance. We construct a GVC knowledge spillovers index by merging data on GVC from the EORA26 dataset with R&D of the trade partner. Results show positive association between the GVC knowledge spillovers index and innovation measured by resident patent per capita. Likewise, we show that trade policy, intellectual property rights’ agreements, and competition policy constitute moderating factors in the nexus between GVC participation and innovation. Our results remain robust when we use alternative measures for our two variables of interest and when we control for foreign knowledge spillovers in imported final goods.

**Supplementary Information:**

The online version contains supplementary material available at 10.1007/s40812-022-00255-9.

## Introduction

In recent years, the mounting trend of global value chains (GVC) participation has slowed due to global investments accompanied with the absence of major liberalization initiatives (World Bank, 2020). The “slowbalization” wave is further augmented by the aftermath of COVID-19 pandemic crisis witnessing deliberate decoupling to unbind the interdependence between industries and countries and therefore prevent the domino effect stirring in crises (Coveri et al., 2020). In this respect, studying the benefits of outsourcing at the country level is crucial to scrutinizing the tradeoff of “reshoring” activities. Beside the conventional theories emphasizing the gains of GVC participation in terms of trade (Baldwin, 2013; Feenstra & Hanson, 1996; Grossman & Rossi-Hansberg, 2008), trade in value added is indeed advantageous in terms of other facets. This paper analyzes the association between GVC participation and countries’ innovation.

While the nexus between trade in final goods and innovation has been examined (Ackigit & Melitz, 2021; Alessandria et al., 2021; Keller, 2004), GVC participation is also likely to have a knowledge driven effect. Indeed, backward participation linkages to GVC transmit embedded foreign knowledge to destination countries that can be signaled by countries’ innovation performance. Aslam et al. (2018) argue that, between 1995 and 2003, foreign knowledge enhanced productivity growth by 0.4% and the former led to more than doubling domestic productivity in developing countries between 2004 and 2014. Undeniably, the gains of international fragmentation of production in terms of technological spillovers are still subject to empirical exploration. While our study highlights the relation between backward linkages to GVC participation and innovation, results emphasize the potential prospect for developing countries in realizing innovation driven economic growth.^1^

Using the simple^2^ offshoring definition, we synthesize the gains of GVC participation in terms of innovation by empirically estimating the association between GVC knowledge spillovers and resident patent per capita. In addition, auxiliary interfering factors in the GVC learning effect are empirically explored namely business environment, institutions, trade policy, competition policy, as well as intellectual property rights’ (IPRs) agreements. Indeed, foreign knowledge spillovers are particularly central for developing countries disadvantaged in technology production. On a flipside however, the learning effect of GVC participation is constrained by prevalent mitigating conditions. First, developing countries are underprivileged with rule of law as a subfactor of institutions’ quality. Second, strengthening IPRs through Trade Related Aspects of Intellectual Property Rights (TRIPS)^3^ trade agreement is argued to be biased towards higher income countries exporting technology. Third, unapt non-tariff trade costs in developing countries discourages foreign exporters of technology (UNCTAD, 2022) and consequently hinders foreign knowledge spillovers. Fourth, lax competition policy disincentivizes innovation (Goto, 2009). Against this background, disentangling the impact of the stated preconditions is crucial to pledging the learning effect of GVC participation. We contribute to the existing literature by studying the multifactorial mitigating dynamism, which is novel to the empirically reviewed nexus between GVC and innovation. Results show a positive and significant relationship between the GVC knowledge spillovers index and domestic innovation. Moreover, we show that trade policy, competition policy, and IPRs agreements constitute a pile of interfering preconditions in the nexus between GVC participation and innovation. Our results remain robust when we use alternative measures for our two variables of interest.

This paper is composed of five sections structured as follows: Sect. 2 reviews the literature on GVC and innovation. Section 3 presents the econometric specification and describes the data. Section 4 is dedicated to the empirical results of the relationship between GVC knowledge spillovers and resident patent per capita in a panel of 83 countries over a time span of 30 years. Section 5 concludes and offers policy implications to the end of fostering innovation particularly in lower-middle income countries.

## Literature review

The relationship between GVC knowledge spillovers and domestic innovation is addressed by blending two strands of literature. The first strand summarizes the association between GVC and domestic innovation, whereas the second strand is related to measuring knowledge spillovers and endogenizing innovation.

A wide strand of the literature focuses on value creation through trade in intermediate goods (Aichele & Heiland, 2018; Antràs & Chor, 2013; Castellani et al., 2015; Johnson & Noguera, 2012; Lee & Yi, 2018). Beside the decrease in marginal cost resulting from specialization, increased production due to GVC participation can be rationalized by increased productivity resulting from technological changes channeled through imports of intermediate goods (Grossman & Helpman, 1991; Kasahara & Rodrigue, 2008; Schmidt, 1997). Despite the conventional concern of the possible adverse effect of GVC participation on developing countries in terms of the relative wages of low skilled labor (Kaplinsky, 2000; Rodrik, 2018), a number of studies emphasize that trade in intermediate goods generates learning and innovation activities (Gereffi et al., 2005; Giuliani et al., 2005; Schmitz & Knorringa, 2000) leading to technological change. Notably, the transfer of technological knowledge through GVC is governed by the nature of the relationship and the distance among GVC participants (OECD, 2017).

Thus, GVC participation can play a crucial role in international knowledge and innovation sharing. Indeed, industry’s performance in GVC enhances innovation (OECD, 2013a, 2013b) since the quality of products is deliberately upgraded to face the demand of foreign supply chains. However, the estimated positive impact depends chiefly on absorptive capacities of the destination country (Corrado et al., 2013). Primarily, developing countries’ GVC participation is deterred by a handful of obstacles rooted in persistent preconditions and strategic behavior (Bell & Albu, 1999; Schmitz, 2004). Likewise, a noteworthy stream in the literature argues that the degree of upgrading in GVC is endogenous to the nature of home institutions (Barrientos et al., 2016; Kano & Tsang, 2020; Pipkin & Fuentes, 2017; Werner, 2012), and the business environment (Dovis & Zaki, 2020). Arguably, the mitigating effect of weak institutions can eventually be alleviated by gaining knowledge through enhanced GVC participation (Kano, 2018). Fortunately, digitalization has recently facilitated GVC participation particularly in developing countries facing high trade costs and prohibitive conditions (World Bank, 2020).

Importing intermediate goods is a channel for technological change due to the potential for foreign knowledge spillovers (Keller, 2002, 2004). Although knowledge is tacit and difficult to measure, imported value-added embed knowledge that can be mirrored in foreign R&D stock endowed in partner countries that export intermediate goods (Coe & Helpman, 1995; Cowan & Jonard, 2004; Maskell & Malmberg, 1999; Zhang et al., 2020). Empirically, a rich strand of literature examines international knowledge diffusion across countries (Bloom et al., 2013, 2016; Bottazzi & Peri, 2007; Coe & Helpman, 1995; Coe et al., 2009; Eaton & Kortum, 1999; Gong & Keller, 2003; Keller, 2004; Malerba et al., 2013). While few results imply a negative short-run effect of GVC participation on innovation in countries with low absorptive capacity (Pietrobelli, 2008 and Farole & Winkler, 2014), other studies find conflicting results. Indeed, the nexus between GVC and innovation is empirically tested using various cross-sectional regressions for developing countries, such as Gehl Sampath and Vallejo (2018) who find that innovation interacts with GVC to foster learning and technological upgrading at the country level. Similarly, the positive association between GVC participation and innovation is empirically recognized for European countries relying on the World Input Output Database (WIOD) (Tajoli & Felice, 2018).

A comprehensive body of literature endogenized innovation using patent per capita (Bloom et al., 2013; Bottazzi & Peri, 2007; Horowitz & Lai, 1996; Malerba et al., 2013; O’Donoghue & Zweimuller, 2004; Scotchmer & Green, 1990; Tajoli & Felice, 2018). According to the knowledge production function framework, R&D personnel and expenditures are inputs to innovation, whereas patenting is the indicator of knowledge creation (Raghupathi & Raghupathi, 2019). While patenting is a direct innovation measure, it can underestimate knowledge creation for two reasons. First, several goods are unpatentable due to their intangible nature (Corrado et al., 2013). Second, some inventors intentionally follow trade secrets’ strategies as a substitute to patenting aiming at preserving their competitive advantage (Crass et al., 2019). Beside domestic R&D, the literature highlights various explanatory determinants to patenting.

Recent variations in domestic patenting activities across countries is justified by different levels of development, size of country, and R&D (WIPO, 2021). In the same vein, literature on trade and innovation highlights the correlation between trade policy and patents since higher tariff rates for example, negatively affect patents for developed and developing countries alike (Vishwasrao et al., 2007). Likewise, the effect of non-tariff measures (NTMs) is of particular importance in countries of the South where infrastructure deficiency augments trade costs (Beghin et al., 2015). Indeed, a harmonized set of trade policy regulations minimizes mismatches leading to positive externalities’ diffusion of GVC in information, communication, and technology (ICT) goods (Ghodsi et al., 2021).

Although developing countries have a technological change opportunity by absorbing knowledge spillovers resulting from linking economies through GVC integration (Mudambi, 2008), knowledge transmission is constrained by a pile of prevalent conditions (Gallini & Wright, 1990). First, innovation catchup necessitates institutional change (Buckley et al., 2020). The share of locally sourced inputs through foreign investors is largest in countries with strong rule of law (Amendolagine et al., 2019) given that complex products require strong institutions (Karam & Zaki, 2019). Second, appropriate IPRs orchestrate the positive effect of GVC participation on innovation (Ali-Yrkkö & Rouvinen, 2015) as they protect investors’ rights. Third, oil dependence induces mitigating conditions to innovation (Namazi & Mohammadi, 2018) since the economies are highly concentrated in extractive industries with a limited value-added. Fourth, competition incentivizes innovation (Marshall & Parra, 2019) whilst competition legislation and effectiveness are middling in Arab countries (Youssef & Zaki, 2022).

In light of the summarized theoretical and empirical strands of literature, this paper contributes to the recognized research gap in two respects. First, our dataset includes central beneficiaries namely lower-middle and low-income countries that are excluded from previous studies despite their technological disadvantage. Second, our empirical strategy incorporates the multifactorial dynamism interfering in the GVC and innovation nexus, which is novel to the literature.

## Methodology and data

Following Tajoli and Felice (2018), our econometric model estimates the relationship between GVC knowledge spillovers and resident patent per capita using the Feenstra and Hanson (1996) offshoring definition.^4^ To construct our variable of interest, the input output value added tables in EORA26 database^5^ (Johnson, 2018; Lenzen et al., 2012, 2013) is merged with R&D data. Thus, the variable we construct (*GVCRD*) is the foreign value added weighted by R&D stock in origin countries as a share of total knowledge weighted value added. Hence, for each destination (importer) country, value added imported from an origin country is multiplied by the corresponding R&D stock in the origin country. Then, the summation is divided by the total R&D weighted value added including the domestic value added as follows:

1 $${\text{GVCRD}}it = \frac{{\mathop \sum \nolimits_{i}^{t} VA ijt*RDjt}}{{ \left( {\mathop \sum \nolimits_{i}^{t} VA ijt*RDjt} \right) + \left( {DVAit*RDit} \right)}}$$

where *i* is the destination (importing) country, *j* is the origin (exporter) country, *t* is time in years, *VA* is the absorbed foreign value added,^6^*DVA* is the domestic value added, and *RD* is the R&D stock.^7^

**Fig. 1:**
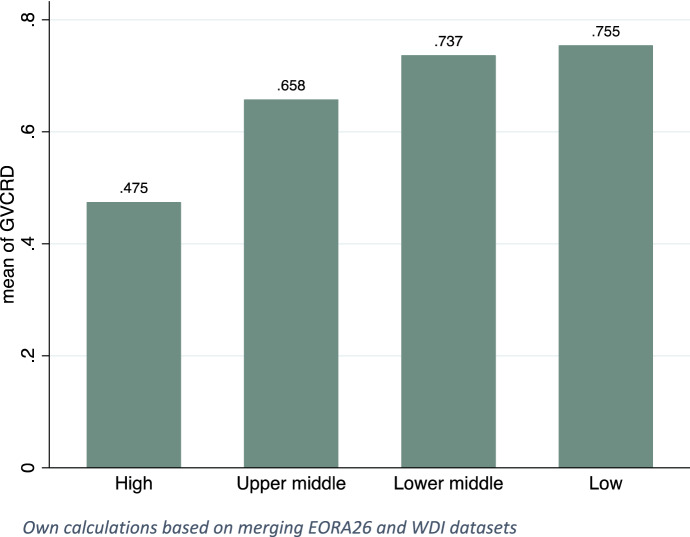
GVC knowledge spillovers index – by income groups*.* Own calculations based on merging EORA26 and WDI datasets

Figure 1 presents the average GVC knowledge spillovers index (GVCRD) in different income groups from 1990 until 2019.^8^ Clearly, the lower the countries’ endowments with domestic R&D stock, the higher the knowledge spillovers through GVC participation. As presented, GVCRD is highest at low-income and decreases at higher levels of income.

**Fig. 2:**
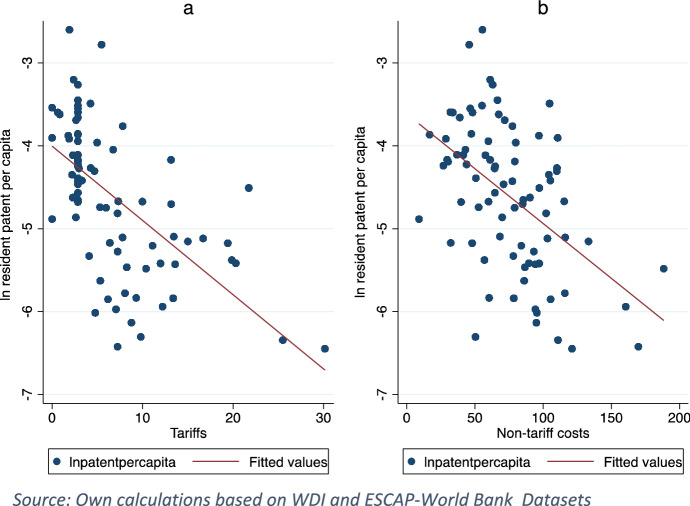
Country average resident patent per capita against trade policy. Source: Own calculations based on WDI and ESCAP-World Bank Datasets

**Fig. 3:**
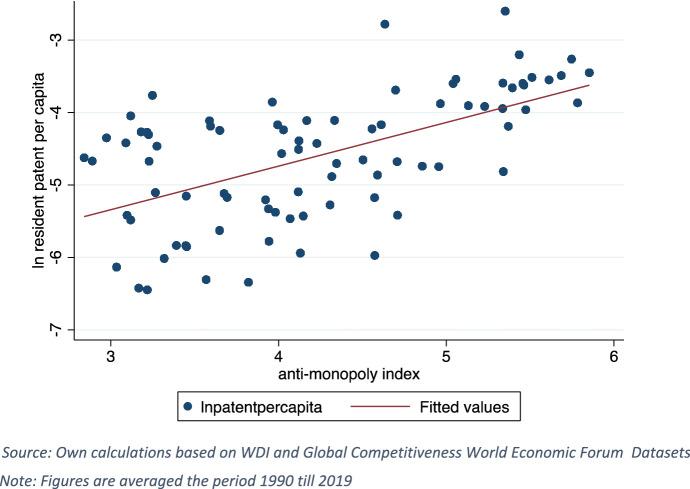
Country average resident patent per capita against competition policy. Source: Own calculations based on WDI and Global Competitiveness World Economic Forum Datasets. Note: Figures are averaged the period 1990 till 2019

Beside testing the hypothesis that enhanced GVC participation is associated with higher domestic innovation due to foreign knowledge spillovers in destination countries, our methodology is extended to capturing a threefold interfering dynamism. First, given the knowledge flows through international trade, tariff and non-tariff trade costs are potential barriers to technology diffusion in destination countries. Figure 2 presents country average resident patent per capita against trade policy. Similar to the negative association between resident patent per capita and tariffs shown in Fig. 2a, b shows an adverse relationship between the former and non-tariff trade costs pointing out to what extent trade policy might matter for domestic innovation. Second, competition policy is a potential vehicle for domestic innovation due to incentivizing investors aiming at preserving market shares. Figure 3 presents country average resident patent per capita against competition proxied by the effectiveness of anti-monopoly law index showing a positive association between the two variables. Third, enforcing IPRs is key in protecting inventions and hence studying its role in fostering domestic innovation is worthwhile. Table 1 presents the average resident patents in accordance with R&D stock level^9^ against TRIPS agreement. Although R&D stock is a chief input to domestic innovation, at high R&D stock^10^ levels, the average of patents is higher in countries involved TRIPS agreement. Likewise, even at low R&D stock, the average of patents is three times higher in countries involved in TRIPS.

**Table 1:** Resident patents averaged against TRIPS

TRIPS	RD stock	
	Low	High
No	1,016.3	19,263.4
Yes	3,224.2	27,414.1

Source: Authors’ own elaboration

Despite the evidenced associations in the data, econometric modeling is crucial to estimating the foreign learning effect of backward GVC participation along with the potential interfering dimensions. Using fixed effects regressions, our contribution to the original model is twofold. First, we rely on the EAORA26 in constructing the GVC knowledge spillovers index allowing for the inclusion of lower-middle and low-income countries. To our knowledge, this is the first paper using the EORA26 dataset in constructing a GVC knowledge spillovers measure. Second, we expand the estimation to include interfering variables in the nexus between GVC and innovation. Equation (2) presents the baseline specification.

2 $${PAT}_{it }= {a}_{0}+ {a}_{1 }{GVCRD}_{it}+ {a}_{2 }{X}_{it}+ {u}_{i}+{u}_{t} + {\varepsilon }_{it}$$

where, ${PAT}_{it}$ is the resident patent per capita in country *i* at year *t* and is expressed in logarithm, *GVCRD*_*it*_ is the GVC knowledge spillovers index in country *i* at year *t*.${X}_{it}$ is a set of control variables including absorptive capacities proxied by domestic R&D stock expressed in logarithm, GDP per capita expressed in logarithm, and total population expressed in logarithm. Tariffs control for trade openness. The share of oil exports in merchandise exports controls for resource dependence,^11^ time to enforce contracts expressed in logarithm proxies business environment, rule of law controls for the quality of institutions, and we control for the interaction between GVCRD and RD stock. ${u}_{i}$ is a time invariant fixed effects vector controlling for cross countries’ unobserved heterogeneity. ${u}_{t}$ is a year fixed effects vector controlling for time-variant heterogeneity. ${\varepsilon }_{it}$ is the error term.

Since we are interested in three GVC learning moderating dimensions, namely trade policy, competition policy, and IPRs, we include a measure of each dimension in the regression framework. In particular, we include non-tariff measures,^12^ the effectiveness of anti-monopoly law as a representative variable for competition policy, and TRIPS^13^ agreement as a measure for IPRs. To address the moderating effect, we interact the representative variable of each dimension with GVCRD.

To untangle the heterogeneous effect of GVC knowledge spillovers in accordance with varying income groups, fixed effects regressions presented in Eq. (2) are repeated while interacting the variable of interest with each income group relying on the World Bank classification definition. Finally, to ensure results’ robustness, a variety of alternative variables are used. First, we use a 1 year lagged R&D expenditures’ weighted GVC^14^ instead of the R&D stock weighted index. Second, backward GVC participation index from TiVA dataset index^15^ is used as the variable of interest instead of the aforementioned *GVCRD*. Third, the dependent variable is altered with non-resident (foreign) patent per capita as a substitute to resident (domestic) patent per capita to guarantee the GVC association with domestic innovation in particular. Fourth, we include a weighted imports R&D measure^16^ as an explanatory variable to the baseline specification to control for knowledge spillovers through final imported goods and distinguish between the latter and the GVC knowledge spillover (Coe & Helpman, 1995).

While our econometric framework captures a robust association between GVC participation and domestic innovation, further research is needed to explicitly address endogeneity between the two variables. Indeed, despite controlling for time and country heterogeneity, reverse causality is possible to the extent that a positive shock to innovation output in the country can expedite foreign markets’ penetration enhancing GVC participation (De Fuentes et al., 2021; Kersan-Škabić, 2019; Tavassoli, 2018).

Based on patents’ data availability, our sample consists of 83 countries from the year 1990 until 2019. Data^17^ relies on the World Development indicators (WDI) to measure the resident and non- resident patent per capita, R&D, GDP per capita, tariffs, as well as oil exports as a percentage of merchandise. Time to enforce contracts comes from the Doing Business dataset whereas rule of law relies on the World Bank World Governance Indicators (WGI) dataset. TRIPS agreement relies on the World Bank Deep Trade Agreements dataset. Non-tariff measures are proxied by the comprehensive non-tariff indicator relying on the ESCAP-World Bank trade costs dataset. Competition is measured by the effectiveness of the anti-monopoly index relying on the Global Competitiveness Index of the World Economic Forum dataset. The alternative backward participation GVC index is the share of foreign value added exported in total value-added exported relying on TiVA dataset for a sample of 57 countries from the year 1995 until 2018. Imports of final goods from the main trading partner and total imports rely on the International Trade Center trade data.

## Empirical results

Results of the association between GVC knowledge spillovers (GVCRD) and resident patent per capita are reported in Tables 2, 3, 4, 5, 6, 7. Table 2 presents the baseline results by gradually introducing control variables in columns (1) to (4). As shown in column (1), there is a direct association between GVCRD and resident patent per capita whilst introducing the main control variables. Results show that GVCRD and domestic R&D stock are two chief innovation inputs evidenced in the positive and significant association between each variable and domestic innovation. All control variables have expected association with domestic innovation. In particular, countries’ absorptive capacity proxied by level of development (GDP per capita) and size (population) are positively associated with resident patent per capita. Fuel exports as a percentage of merchandise exports are negatively associated with domestic innovation showing that oil dependence challenges innovation due to the high concentration in low value-added extracting activities. In addition, the negative association of tariffs rate with resident patent per capita shows that barriers to trade are also likely to hinder technology diffusion. Although higher (lower) tariff rate is partially reflected in lower (higher) absorbed value added, the inclusion of tariffs is relevant for two reasons. First, the variable of interest is a spillover GVC index incorporating non-tariffed intangible^18^ capital. Second, trade in final goods has a learning effect undepicted in the GVC measure. Higher tariffs negatively affect the circulation of these products and thus the learning effect associated to them.

**Table 2:** Baseline regression for the effect of GVCRD on resident patent per capita

	Dependent Variable: Log of Resident Patent per capita			
	(1)	(2)	(3)	(4)
GVCRD	0.256** (0.1)	0.248** (0.102)	0.234** (0.102)	2.812*** (0.326)
Log (RD stock)	0.239*** (0.062)	0.234*** (0.063)	0.238*** (0.063)	0.94*** (0.105)
Log (GDP per capita)	0.548*** (0.086)	0.546*** (0.086)	0.43*** (0.09)	0.42*** (0.088)
Log (population)	0.541*** (0.172)	0.531*** (0.174)	0.648*** (0.175)	0.73*** (0.173)
Fuel exports	− 0.003*** (0.001)	− 0.003*** (0.001)	− 0.003*** (0.001)	− 0.003*** (0.001)
Tariffs	− 0.011*** (0.001)	− 0.011*** (0.001)	− 0.011*** (0.001)	− 0.012*** (0.001)
Log (Time to Contracts)		− 0.061 (0.144)	− 0.016 (0.144)	− 0.051 (0.142)
Rule of Law			0.004*** (0.001)	0.004*** (0.001)
RD*GVCRD				− 0.886*** (0.107)
Constant	− 11.362*** (1.389)	− 11.102*** (1.517)	− 11.865*** (1.52)	− 14.45*** (1.531)
No. of Observations	2490	2490	2490	2490
No. of Countries	83	83	83	83
R^2^	0.146	0.146	0.154	0.178
Country FE	Yes	Yes	Yes	Yes
Year FE	Yes	Yes	Yes	Yes

(i) Standard errors are in parentheses. (ii) Fixed effects are removed for brevity. (iii) ***p < 0.01, **p < 0.05, *p < 0.1

**Table 3:** The effect of trade policy on resident patent per capita

	Dependent Variable: Log of Resident Patent per capita				
	(1)	(2)	(3)	(4)	(5)
GVCRD	2.812*** (0.326)	2.516*** (0.334)	3.623*** (0.394)	2.818*** (0.359)	3.826*** (0.409)
Log (RD stock)	0.94*** (0.105)	0.917*** (0.107)	0.999*** (0.107)	0.967*** (0.109)	1.036*** (0.109)
Log (GDP per capita)	0.42*** (0.088)	0.456*** (0.101)	0.465*** (0.1)	0.459*** (0.101)	0.467*** (0.1)
Log (population)	0.73*** (0.173)	1.042*** (0.201)	0.864*** (0.202)	1.049*** (0.201)	0.876*** (0.202)
Fuel exports	− 0.003*** (0.001)	− 0.003*** (0.001)	− 0.001* (0.001)	− 0.003*** (0.001)	− 0.002* (0.001)
Tariffs	− 0.012*** (0.001)	− 0.02*** (0.002)	− 0.02*** (0.002)	− 0.009* (0.005)	− 0.011** (0.005)
Log (time to contracts)	− 0.051 (0.142)	− 0.157 (0.128)	− 0.304** (0.131)	− 0.149 (0.128)	− 0.293** (0.131)
Rule of Law	0.004*** (0.001)	0.002** (0.001)	0.003*** (0.001)	0.002** (0.001)	0.003*** (0.001)
RD*GVCRD	− 0.886*** (0.107)	− 0.788*** (0.109)	− 0.928*** (0.111)	− 0.865*** (0.114)	− 0.984*** (0.116)
NTMs		− 0.004*** (0.001)	0.003* (0.001)	− 0.004*** (0.001)	0.002* (0.001)
NTMs*GVCRD			− 0.01*** (0.002)		− 0.009*** (0.002)
Tariffs*GVCRD				− 0.015** (0.007)	− 0.012* (0.007)
Constant	− 14.45*** (1.531)	− 15.927*** (1.744)	− 15.056*** (1.741)	− 16.244*** (1.748)	− 15.336*** (1.746)
No. of Observations	2490	2050	2050	2050	2050
No. of Countries	83	82	82	82	82
R-squared	0.178	0.189	0.2	0.191	0.191
Country FE	Yes	Yes	Yes	Yes	Yes
Year FE	Yes	Yes	Yes	Yes	Yes

(i) Standard errors are in parentheses. (ii) Fixed effects are removed for brevity. (iii) ***p < 0.01, **p < 0.05, *p < 0.1. (iv) NTMs are total trade costs with the main trade partner excluding tariffs

**Table 4:** The effect of competition policy on resident patent per capita

	Dependent Variable: Log of Resident Patent per capita				
	(1)	(2)	(3)	(4)	(5)
GVCRD	2.812*** (0.326)	2.337*** (0.353)	2.447*** (0.351)	2.827*** (0.38)	2.745*** (0.379)
Log (RD stock)	0.94*** (0.105)	0.853*** (0.112)	0.849*** (0.111)	0.771*** (0.114)	0.797*** (0.114)
Log (GDP per capita)	0.42*** (0.088)	0.473*** (0.115)	0.485*** (0.115)	0.43*** (0.116)	0.457*** (0.115)
Log (population)	0.73*** (0.173)	0.882*** (0.212)	0.986*** (0.211)	0.959*** (0.213)	1.024*** (0.212)
Fuel exports	− 0.003*** (0.001)	− 0.003*** (0.001)	− 0.003*** (0.001)	− 0.003*** (0.001)	− 0.003*** (0.001)
Tariffs	− 0.012*** (0.001)	− 0.021*** (0.002)	− 0.02*** (0.002)	− 0.021*** (0.002)	− 0.02*** (0.002)
Log (time to contracts)	− 0.051 (0.142)	− 0.099 (0.132)	− 0.033 (0.131)	− 0.03 (0.133)	0.003 (0.132)
Rule of law	0.004*** (0.001)	0.003*** (0.001)	0.003*** (0.001)	0.003*** (0.001)	0.003*** (0.001)
RD*GVCRD	− 0.886*** (0.107)	− 0.702*** (0.115)	− 0.728*** (0.115)	− 0.646*** (0.116)	− 0.69*** (0.116)
Anti-Monopoly		− 0.024 (0.019)	0.904*** (0.175)	0.079** (0.036)	0.868*** (0.176)
(Anti-Monopoly)^2^			− 3.705*** (0.696)		− 3.304*** (0.721)
Anti-Monopoly*GVCRD				− 0.178*** (0.052)	− 0.112** (0.054)
Constant	− 14.45*** (1.531)	− 15.075*** (1.842)	− 12.435*** (1.895)	− 15.802*** (1.849)	− 13.179*** (1.927)
No. of observations	2490	2075	2075	2075	2075
No. of Countries	83	83	83	83	83
R^2^	0.178	0.166	0.178	0.171	0.18
Country FE	Yes	Yes	Yes	Yes	Yes
Year FE	Yes	Yes	Yes	Yes	Yes

(i) Standard errors are in parentheses. (ii) Fixed effects are removed for brevity. (iii) ***p < 0.01, **p < 0.05, *p < 0.1. (iv) Anti-Monopoly is measured by the effectiveness of the anti-monopoly law index

**Table 5:** The effect of IPRs agreements on resident patent per capita

	Dependent Variable: Log of Resident Patent per capita			
	(1)	(2)	(3)	(4)
GVCRD	2.812*** (0.326)	2.796*** (0.326)	2.755*** (0.325)	2.823*** (0.338)
Log (RD stock)	0.94*** (0.105)	0.942*** (0.105)	0.942*** (0.104)	0.961*** (0.107)
Log (GDP per capita)	0.42*** (0.088)	0.455*** (0.09)	0.449*** (0.09)	0.438*** (0.091)
Log (population)	0.73*** (0.173)	0.729*** (0.173)	0.846*** (0.174)	0.825*** (0.177)
Fuel exports	− 0.003*** (0.001)	− 0.003*** (0.001)	− 0.004*** (0.001)	− 0.004*** (0.001)
Tariffs	− 0.012*** (0.001)	− 0.012*** (0.001)	− 0.011*** (0.001)	− 0.011*** (0.001)
Log (time to contracts)	− 0.051 (0.142)	− 0.059 (0.142)	− 0.015 (0.142)	− 0.02 (0.142)
Rule of law	0.004*** (0.001)	0.004*** (0.001)	0.004*** (0.001)	0.004*** (0.001)
RD*GVCRD	− 0.886*** (0.107)	− 0.88*** (0.107)	− 0.856*** (0.106)	− 0.89*** (0.116)
TRIPS		− 0.037* (0.02)	− 0.335*** (0.07)	− 0.358*** (0.077)
TRIPS*low-excluded			0.318*** (0.072)	0.317*** (0.072)
TRIPS*GVCRD				0.041 (0.057)
Constant	− 14.45*** (1.531)	− 14.552*** (1.531)	− 15.464*** (1.539)	− 15.293*** (1.557)
No. of Observations	2490	2490	2490	2490
No. of Countries	83	83	83	83
R^2^	0.178	0.179	0.186	0.186
Country FE	Yes	Yes	Yes	Yes
Year FE	Yes	Yes	Yes	Yes

(i) Standard errors are in parentheses. (ii) Fixed effects are removed for brevity. (iii) ***p < 0.01, **p < 0.05, *p < 0.1. (iv) TRIPS is a dummy variable equals 1 if the country signs a deep trade agreement involving intellectual property rights and equals zero otherwise

**Table 6:** The effect of GVCs on innovation in different income groups

	Dependent Variable: Log of Resident Patent per capita	
	(1)	(2)
GVCRD	2.812*** (0.326)	1.952*** (0.459)
Log (RD Stock)	0.94*** (0.105)	0.738*** (0.116)
Log (GDP per capita)	0.42*** (0.088)	0.402*** (0.088)
Log (Population)	0.73*** (0.173)	0.628*** (0.179)
Fuel Exports	− 0.003*** (0.001)	− 0.003*** (0.001)
Tariffs	− 0.012*** (0.001)	− 0.011*** (0.001)
Log (Time to Contracts)	− 0.051 (0.142)	− 0.069 (0.142)
Rule of Law	0.004*** (0.001)	0.004*** (0.001)
RD*GVCRD	− 0.886*** (0.107)	− 0.756*** (0.119)
GVCRD*UpperMiddle		0.189 (0.217)
GVCRD*LowerMiddle		0.852*** (0.237)
GVCRD*LowIncome		0.999 (1.215)
Constant	− 14.45*** (1.531)	− 12.92*** (1.612)
No. of Observations	2490	2490
No. of Countries	83	83
R-squared	0.178	0.184
Country FE	Yes	Yes
Year FE	Yes	Yes

(i) Standard errors are in parentheses. (ii) Fixed effects are removed for brevity. (iii) ***p < 0.01, **p < 0.05, * p < 0.1

**Table 7:** All explanatory variables

	Dependent Variable: Log of Resident Patent per capita		
	(1)	(2)	(3)
GVCRD	2.812*** (0.326)	2.537*** (0.342)	4.246*** (0.44)
Log (RD Stock)	0.94*** (0.105)	0.893*** (0.108)	0.959*** (0.112)
Log (GDP per capita)	0.42*** (0.088)	0.534*** (0.116)	0.535*** (0.117)
Log (population)	0.73*** (0.173)	1.358*** (0.212)	1.246*** (0.216)
Fuel exports	− 0.003*** (0.001)	− 0.004*** (0.001)	− 0.003*** (0.001)
Tariffs	− 0.012*** (0.001)	− 0.018*** (0.002)	-0.008 (0.006)
Log (time to contracts)	− 0.051 (0.142)	− 0.031 (0.128)	− 0.147 (0.131)
Rule of law	0.004*** (0.001)	0.001 (0.001)	0.002* (0.001)
RD*GVCRD	− 0.886*** (0.107)	− 0.77*** (0.112)	− 0.921*** (0.12)
NTMs		− 0.004*** (0.001)	0.003** (0.001)
Anti-Monopoly		1.049*** (0.171)	0.882*** (0.172)
(Anti-Monopoly)^2^		− 4.291*** (0.678)	− 3.427*** (0.706)
TRIPS		− 0.289*** (0.069)	− 0.347*** (0.081)
TRIPS*low-excluded		0.317*** (0.071)	0.403*** (0.072)
Tariffs*GVCRD			− 0.013* (0.007)
NTMs*GVCRD			− 0.011*** (0.002)
Anti-Monopoly*GVCRD			− 0.108** (0.054)
TRIPS*GVCRD			− 0.014 (0.065)
Constant	− 14.45*** (1.531)	− 14.464*** (1.88)	− 15.247*** (1.933)
No. of observations	2490	2050	2050
No. of countries	83	82	82
R^2^	0.178	0.215	0.231
Country FE	Yes	Yes	Yes
Year FE	Yes	Yes	Yes

(i) Standard errors are in parentheses. (ii) Fixed effects are removed for brevity. (iii) ***p < 0.01, **p < 0.05, *p < 0.1. (iv) NTMs are total trade costs with the main trade partner excluding tariffs. Anti-Monopoly is effectiveness of the anti-monopoly law index. TRIPS is a dummy variable equals 1 if the country signs a deep trade agreement involving intellectual property rights and equals zero otherwise.

As additional control variables are introduced, column (2) shows an insignificant association between time to enforce contracts whereas column (3) shows a positive relationship between the quality of institutions (rule of law) and domestic innovation. The interaction term between domestic R&D stock and GVCRD is introduced in column (4) showing substitutability between foreign and domestic knowledge as innovation inputs (Coe & Helpman, 1995). Yet, the net effect of GVCRD is positive and higher in magnitude when the interaction term is introduced. At the average level of R&D stock, a 0.01 increase in GVCRD is associated with 0.23% increase in resident patent per capita, whereas at the minimum level of R&D stock, a 0.01 increase in GVCRD is associated with a 2% increase in resident patent per capita. Column (4) reports the baseline results and is presented as column (1) in all tables thereafter for comparison.

To further explore the relevance of trade policy, Table 3 presents the results whilst introducing non-tariff measures (NTMs). Although both tariffs and NTMs increase trade costs, the latter can have positive association with domestic innovation due the bounded standardization and licensing requirements. However, consistent with tariffs’ direct negative association with domestic innovation shown in column (1), column (2) shows that NTMs and resident patent per capita are negatively associated with a smaller magnitude compared to tariffs. Likewise, while both tariffs and NTMs negatively interact with GVCRD as presented in columns (3) and (4), the net effect of GVCRD is consistently positive and the magnitude of the coefficient increases when NTMs interaction with GVCRD is introduced. Moreover, it is worthy to note that time to enforce contracts shows negative association with domestic innovation as NTMs are introduced as the latter are augmented by domestic regulations including certification, licensing, and contractual procedures. Column(5) shows consistency as both tariffs and NTMs interaction with GVCRD are controlled for in one regression. Results show that at the average levels of R&D stock, tariffs, and NTMs, a 0.01 increase in GVCRD is associated with a 0.18% increase is resident patent per capita.

In addition to trade policy, competition is another vital policy dimension to domestic innovation. Results of introducing the effectiveness of anti-monopoly law index as a *de jure* measure of competition to the baseline specification are presented in Table 4. Although column (2) shows an insignificant direct association, column (3) reports an inverted U-shaped relationship between competition and resident patent per capita. Empirically, the effect of competition on domestic innovation is complex, non-linear, and unexpectedly changes (Aghion et al., 2005). From a theoretical standpoint, as the *de jure* competition index increases, inventors (leaders) expect new entrants and hence engage in patenting to protect their inventions. Yet, a competition driven increase in patents is unguaranteed for two reasons. First, innovation is spatially concentrated in high income countries and new entry is endogenous to absorptive capacities (Corrado et al., 2013). Indeed, given low capacities, frontier technologies can be inappropriate to absorb by developing countries. Second, alternative to patenting, leaders can choose to engage in trade secrets to protect their monopoly power at higher competition (Crass et al., 2019). Put differently, in light of fierce competition, using patents as an innovation index may underestimate innovation performance. Results in column (4) shows a dampening moderating GVC learning effect of competition. As the *de jure* competition index increases, the GVCRD positive association with resident patent per capita decreases. Yet, the net GVCRD association remains positive and the results are consistent when both the interaction and the squared term are included in one regression as reported in column (5). At the average level of R&D stock and the competition index, a 0.01 increase in GVCRD is associated with a 0.87% increase in resident patent per capita.

In light of the conceptual relevance of enforcing IPRs to incentivizing innovators, Table 5 presents the results of the direct and moderating learning effect of TRIPS agreement. Results reveal an advantage reallocation of IPRs enforcement in terms of domestic innovation against low-income countries. Although column (2) signals an adverse association, column (3) shows that TRIPS agreement exerts a positive effect when interacted with the sample of countries excluding low income. Notwithstanding the innovation incentivizing role of IPRs, the latter increases the cost of imitation on disadvantaged laggards. Due to the increasing returns of technology adoption (Acemoglu, 2002), technology leaders (high income countries) with higher innovation status quo have a lower cost of technology adoption and can therefore foster innovation easier than less advantaged laggards (low-income countries). Despite its negative effect, TRIPS agreement is not adversely moderating the GVC knowledge spillovers as evidenced in the insignificant interaction between the former and GVCRD in column (4).^19^ Clearly, the magnitude of the moderating effect of IPRs is conditional on the mode and complexity of GVC participation. However, our results are limited to the simple backward GVC participation mode aggregating the share of foreign value added absorbed for all sectors.

Table 6 presents the results of GVCRD interaction with different income groups to disentangle the GVC knowledge spillovers effect in correspondence with varying income levels. Column (2) provides evidence on two main theoretical foundations. First, the backwardness effect (Aghion & Howitt, 2007) is shown in the interaction between GVCRD and lower middle-income group. In reference to high-income, lower middle-income countries have the highest positive association between GVCRD and resident patent per capita. Indeed, countries at earlier stages of development benefit more from knowledge spillovers than developed counterparts. Second, knowledge spillovers require a threshold of minimum absorptive capacity (Falvey et al., 2007). As shown in the insignificant interaction between low-income countries and GVCRD, GVC knowledge spillovers are mitigated by the lesser absorptive capacity.^20^

Results of combining all explanatory variables in one regression are reported in Table 7. The direct effect of the three explored dimensions is presented in column (2). First, regarding trade policy, both explicit (tariffs) and implicit (non-tariff) trade protection have a direct negative association with domestic innovation with a lesser magnitude of the latter. Second, competition and domestic innovation are positively related till a maximum threshold beyond which increasing competition is negatively associated with domestic innovation. Third, the positive association between IPRs and domestic innovation is conditional on excluding low-income countries evidencing a bias of IPRs enforcement in laggard low-income economies. Showing consistency with previous individual results, column (3) combines the GVC learning moderating effect of each dimension along with the direct effect in one regression.

Across a variety of checks presented in Table 8, GVCRD positive association with resident patent per capita remains robust. Alternative to weighting value added with R&D stock, column (2) shows the results of weighting the value added absorbed with subsequent R&D expenditures in source countries. Similar to the R&D stock weighted spillover GVC, the R&D expenditures weighted index is positively associated with resident patent per capita. As another check, column (3) presents the results of altering the simple backward linkages to GVC (GVCRD) with a more complex backward GVC participation index. Consistent with simple offshoring GVC definition, the share of foreign value-added exported index from TiVA dataset is positively associated with resident patent per capita. The complex GVC measure already captures a learning effect as it entails that foreign inputs cross borders more than once. Likewise, all control variables preserve the baseline results’ sign and significance. Column (4) reports the results of altering the dependent variable with a substitute innovation measure being it the non-resident patent per capita. The insignificance of GVCRD coefficient reflects that GVC knowledge spillovers matters for domestic rather than foreign patents. Finally, column (5) presents the results when foreign knowledge spillovers through imported final goods is controlled for. In line with the literature, knowledge transmission in imported goods (Coe & Helpman, 1995) may dilute the captured GVCRD effect. Yet, results show a consistent GVCRD association after controlling for imports’ knowledge spillovers. The four robustness checks employed confirm the positive association between GVC participation and domestic innovation.

**Table 8:** Robustness checks

Dependent variable	Log of resident patent per capita			Log of non-resident patent per capita	Log of resident patent per capita
	Baseline (1)	RD exp. weight (2)	TiVA (3)	(4)	(5)
GVCRD	2.812*** (0.326)	0.082*** (0.031)	0.046*** (0.005)	0.483 (1.023)	2.872*** (0.32)
RD	0.94*** (0.105)	0.264*** (0.029)	1.042*** (0.101)	− 0.135 (0.329)	1.118*** (0.107)
Log (GDP per capita)	0.42*** (0.088)	0.343*** (0.09)	1.187*** (0.105)	1.406*** (0.278)	0.309*** (0.088)
Log (Population)	0.73*** (0.173)	0.528*** (0.166)	2.716*** (0.213)	5.626*** (0.543)	0.549*** (0.172)
Fuel Exports	− 0.003*** (0.001)	− 0.003*** (0.001)	− 0.007*** (0.002)	0.003 (0.003)	− 0.011*** (0.001)
Tariffs	− 0.012*** (0.001)	− 0.019*** (0.001)	− 0.019*** (0.005)	− 0.026*** (0.004)	− 0.002** (0.001)
Log (Time to Contracts)	− 0.051 (0.142)	0.029 (0.139)	0.132 (0.12)	− 0.159 (0.445)	0.036 (0.14)
Rule of Law	0.004*** (0.001)	0.005*** (0.001)	0.011*** (0.003)	0.003 (0.003)	0.005*** (0.001)
RD*GVCRD	− 0.886*** (0.107)	− 0.043* (0.024)	− 0.024*** (0.003)	− 0.474 (0.335)	− 0.945*** (0.105)
IMRD					0.4*** (0.052)
RD*IMRD					− 0.125*** (0.019)
Constant	− 14.45*** (1.531)	− 10.178*** (1.421)	− 69.622*** (4.152)	− 54.645*** (4.808)	− 13.57*** (1.509)
No. of Observations	2490	2340	1368	2490	2460
No. of Countries	83	81	57	83	82
R^2^	0.178	0.214	0.429	0.145	0.204
Country FE	Yes	Yes	Yes	Yes	Yes
Year FE	Yes	Yes	Yes	Yes	Yes

(i) Standard errors are in parentheses. (ii) Fixed effects are removed for brevity. (iii) *** p < 0.01, ** p < 0.05, * p < 0.1. (iv) GVCRD in columns (1) and (4) is RD stock weighted GVC. GVCRD in column (2) is RD expenditures weighted GVC lagged one year. GVCRD in column (3) is the backward GVC index in TiVA dataset. RD in columns (1) and (4) is the log of RD Stock. (v) RD in columns (2) and (3) is the RD expenditures as a percentage of GDP. (vi) IMRD is a foreign R&D weighted imports measure (Coe & Helpman, 1995) computed by multiplying R&D stock of main trading partner by the share of imports from main trading partner in total imports in the destination country

In summary, empirical results show that backward participation linkages to GVC is positively associated with resident patent per capita particularly in lower-middle income countries. Grounded on the presented results, we argue the following: First, backward GVC participation is accompanied with technological change due to foreign knowledge spillovers. Second, the quality of institutions matters to domestic innovation reflected in the persistent positive and significant effect of rule of law. Third, both tariff and non-tariff costs matter for domestic innovation. Fourth, the positive association between competition and domestic innovation is non-monotonic. Fifth, IPRs incentivize domestic innovation with a bias against low-income countries.

## Conclusion

By emphasizing the relevance of GVC participation as a channel for fostering domestic innovation, we draw several conclusions. We show that the GVC knowledge spillovers index we construct is positively associated with resident patent per capita and that lower middle-income countries are the chief beneficiaries of a backward GVC driven innovation. We also synthesize the interfering direct and moderating effects of several dimensions with the GVC innovation nexus. In particular, we show that trade policy, competition policy, and IPRs enforcement are directly associated with resident patent per capita. Although tariff and non-tariff costs dampen GVC knowledge spillovers, the net effect of the latter is consistently positive. We also conclude that the direct positive association between enhancing competition and resident patent per capita is indeterministic due to the captured inverted U-shaped relationship. Likewise, IPRs enforcement incentivize innovation with a bias against low-income countries.

This study contributes to the post COVID-19 controversial discourse on the trade-off of reshoring activities by evidencing the opportunity cost of decoupling in terms of domestic innovation. From a policy standpoint, the positive and significant association between GVC and innovation advocates encouraging backward linkages to GVC particularly in lower-middle income countries exhibiting the highest positive effect of GVC knowledge spillovers. To this end, recommended policies to fostering the learning effect of GVC participation in developing countries are fivefold. First, lowering unnecessary trade costs (implied by both tariffs and non-tariff measures) is key to encouraging foreign exporters of intermediate goods. Second, policies targeting institutions’ evolution and rule of law promotion are compulsory to fostering domestic innovation. Third, negotiations of deep trade agreements involving property rights are central to guarantying unbiases against low-income countries disadvantaged in technology production. Fourth, enhancing competition in countries with lax competition policy should be cautiously implemented due to the non-linearity of the relationship. Fifth, fostering the absorptive capacity in low-income countries by investing in human and physical capital is necessary to realizing a GVC driven technological change. The evidence-based policies provided by this paper paves to the ninth global goal^21^ of the United Nations sustainable development goals (SDGs) intended to be achieved by the year 2030.

## Supplementary Information

Below is the link to the electronic supplementary material.

Supplementary file1 (DOCX 38 KB)

## Data Availability

Raw data were generated from EORA dataset and the World Development Indicators dataset. Derived data supporting the findings of this study are available from the corresponding author Chahir Zaki on request.

## Footnotes

1 Economic growth is either factor driven, investment driven, or innovation driven (Raghupathi and Raghupathi, 2019). The share of innovation driven growth accounts to more than 50% of economic growth drivers (Kayal, 2008; OECD, 2005).

2 The simple definition is limited to intermediate goods’ crossing borders at least once. The complex definition of vertical specialization guarantees the reexporting of intermediate imports.

3 Article 31 in the amended agreement provides WTO members with special licenses to produce and export medicines to other members having insufficient domestic production.

4 Share of foreign value added absorbed through imported intermediate goods to the total value added of intermediate goods including the domestic value added.

5 The database includes 189 countries from 1990 till 2019. All countries are aggregated to a common 26 sector classification. Appendix 1 in supplementary material presents a list of the aggregated sectors.

6 Using the UN Systems of National Accounts 1993, VA is measured by compensation of employees, taxes on production, subsidies on production, net operating surplus, net mixed income, and consumption of fixed capital. https://worldmrio.com/eora26/.

7 Number of researchers working in R&D per million of the population.

8 Appendix 2 in supplementary material presents the list of countries and the corresponding income group.

9 Low RD stock is lower than the median value. High RD stock is equal to or greater than the median value.

10 R&D stock is also a measure of closeness to world technological frontier (Tajoli & Felice, 2018).

11 Oil dependence constrains innovation because economies are concentrated in low value-added extracting activities (Namazi & Mohammadi, 2018).

12 Measured by the non-tariff trade costs with the main trading partner.

13 Dummy variable equals 1 if the country signed an agreement involving TRIPS and 0 otherwise.

14 To measure this variable, we alternate R&D stock in Eq. (1) with R&D expenditures as a percentage of GDP.

15 It is a complex GVC measure entailing that foreign value-added cross borders more than once. GVCRD is a measure of foreign value-added absorbed regardless of whether it is exported or not.

16 This is measured by multiplying R&D stock of the main trading partner with the imports of goods from the main trading partner divided by total imports in destination.

17 Appendix 3 in supplementary material presents variables’ definitions and descriptive statistics.

18 Refers to brand, design, science and technology constituting more than 30% of total GVC capital on average (WIPO, 2017).

19 Appendix 4 in supplementary material presents a heterogenous negative moderating effect of WIPO. Although both IPRs agreements aim at incentivizing innovation, TRIPS is more effective for two reasons. First, TRIPS is a binding agreement while WIPO specifies non-binding mediation in resolving disputes. Second, TRIPS provide flexibility of regulations to governments unlike WIPO standardizing a one size fits all regulations’ system (McCalman, 2005).

20 Low-income countries have the least R&D stock signaling the extent of low human and physical capital accumulation.

21 SDG 9 aims at building resilient infrastructure, promoting industrialization, and fostering innovation.

## References

[CR1] Acemoglu D (2002). Directed technical change. The Review of Economic Studies.

[CR2] Ackigit U, Melitz M (2021). Innovation and trade. Handbook of international economics.

[CR3] Aghion P, Bloom N, Blundell R, Griffith R, Howitt P (2005). Competition and innovation: An Inverted-U Relationship. The Quarterly Journal of Economics.

[CR4] Aghion P, Howitt P (2007). Capital, innovation, and growth accounting. Oxford Review of Economic Policy.

[CR5] Aichele R, Heiland I (2018). Where is the value added? Trade liberalization and production Networks. Journal of International Economics.

[CR6] Alessandria GA, Arkolakis C, Ruhl KJ (2021). Firm dynamics and trade. Annual Review of Economics.

[CR7] Ali-Yrkkö J, Rouvinen P (2015). Slicing up global value chains: A micro view. Journal of Industry, Competition, and Trade.

[CR8] Amendolagine V, Presbitaro A, Rabellotti R, Sanfillipo M (2019). Local sourcing in developing countries: The role of foreign direct investment and global value chains. World Development.

[CR9] Antràs P, Chor D (2013). Organizing the global value chain. Econometrica.

[CR10] Aslam A, Eugster J, Ho G, Jaumotte F, Osorio-Buitron C, Piazza R (2018). Globalization helps spread knowledge and technology across borders, IMF Blog.

[CR11] Baldwin R (2013). Global supply chains: why they emerged, why they Matter, and where they are going? Global Value Chains in a Changing World.

[CR12] Barrientos S, Knorringa P, Evers B, Visser M, Opondo M (2016). Shifting regional dynamics of global value chains: Implications for economic and social upgrading in African horticulture. Environment and Planning.

[CR13] Beghin J, Maertens M, Swinnen J (2015). Nontariff measures and standards in trade and global value chains. The Annual Review of Resource Economics.

[CR14] Bell M, Albu M (1999). Knowledge systems and technological dynamism in industrial clusters in developing countries. World Development.

[CR15] Bloom N, Drake M, Reneen J (2016). Trade induced technical change? The impact of Chinese imports on innovation, IT, and productivity. The Review of Economic Studies.

[CR16] Bloom N, Schankerman M, Reenen J (2013). Identifying technology spillovers and product market rivalry. Econometrica.

[CR17] Bottazzi L, Peri G (2007). The international dynamics of R&D and innovation in the long run and in the short run. The Economic Journal.

[CR18] Buckley J, Strange R, Timmer M, de Vries G (2020). Catching up in the global factory: Analysis and policy implications. Journal of International Business Policy.

[CR19] Castellani D, Mancusi L, Santangelo D, Zanfei A (2015). Exploring the links between offshoring and innovation. Journal of Industrial and Business Economics.

[CR20] Coe T, Helpman E (1995). International R&D spillovers. European Economic Review.

[CR21] Coe T, Helpman E, Hoffmaister A (2009). International R&D spillovers and Institutions. European Economic Review.

[CR22] Corrado C, Haskel J, Jona-Lasinio C, Iommi M (2013). Innovation and intangible investment in Europe, Japan and the United States. Oxford Review of Economic Policy.

[CR23] Coveri A, Cozza C, Nascia L, Zanfei A (2020). Supply chain contagion and the role of industrial policy. Journal of Industrial and Business Economics.

[CR24] Cowan R, Jonard N (2004). Network structure and the diffusion of knowledge. Journal of Economic Dynamics and Control.

[CR25] Crass D, Valero F, Pitton F, Ramer C (2019). Protecting innovation through patents and trade secrets: Evidence for firms with a single innovation. International Journal of the Economics and Business.

[CR26] De Fuentes C, Niosi J, Ara Peerally J (2021). Exploring innovation and export interplay in Canadian firms. Economics of Innovation and New Technology.

[CR27] Dovis M, Zaki C (2020). Global value chains and local business environment: which factors do really matter in developing countries?. Review of Industrial Organization.

[CR28] Eaton J, Kortum S (1999). International technical diffusion: Theory and measurement. International Economic Review.

[CR02] Falvey R, Foster N, Greenaway D (2007). Relative backwardness, absorptive capacity and knowledge spillovers. Economics Letters.

[CR29] Farole T, Winkler D (2014). Making foreign direct investment work for Sub-Saharan Africa: Local spillovers and competitiveness in global value chains. Directions in development-Trade.

[CR30] Feenstra R, Hanson G (1996). Globalization, outsourcing and wage inequality. American Economic Review.

[CR31] Gallini N, Wright B (1990). Technology transfer under asymmetric information. Rand Journal of Economics.

[CR33] GehlSampath P, Vallejo B (2018). Trade, global value chains and upgrading: What, when and how?. The European Journal of Development Research.

[CR34] Gereffi G, Humphrey J, Sturgeon T (2005). The governance of global value chains. Review of International Political Economy.

[CR35] Ghodsi, M., Adarov, A., Exadaktylos, D., Stehrer, R., & Stöllinger, R. (2021). Production and trade from an EU Perspective. Report 456. *The Vienna Institute for International Economic Studies.*

[CR36] Giuliani E, Pietrobelli C, Rabellotti R (2005). Upgrading in global value chains: Lessons from Latin American clusters. World Development.

[CR37] Gong G, Keller W (2003). Convergence and polarization in global income levels: A review of recent results on the role of international technology diffusion. Research Policy.

[CR38] Goto A (2009). Innovation and competition policy. The Japanese Economic Review.

[CR39] Grossman G, Helpman E (1991). Innovation and growth in the global economy.

[CR40] Grossman M, Rossi-Hansberg E (2008). Trading tasks: A simple theory of offshoring. American Economic Review.

[CR42] Horowitz A, Lai E (1996). Patent length and the rate of innovation. International Economic Review.

[CR45] Johnson R (2018). Measuring global value chains. Annual Review of Economics.

[CR46] Johnson R, Noguera G (2012). Accounting for intermediates: Production sharing and trade in value added. Journal of International Economics.

[CR47] Kano L (2018). Global value chain governance: A relational perspective. Journal of International Business Studies.

[CR48] Kano L, Tsang E (2020). Global value chains: A review of the multidisciplinary literature. Journal of International Business Studies.

[CR49] Kaplinsky R (2000). Globalisation and unequalisation: What can be learned from value chain analysis?. The Journal of Development Studies.

[CR01] Karam F, Zaki C (2019). Why Can’t MENA Countries Trade More? The Curse of Bad Institutions. Quarterly Review of Economics and Finance.

[CR50] Kasahara H, Rodrigue J (2008). Does the use of imported intermediates increase productivity? Plant-level evidence. Journal of Development Economics.

[CR51] Kayal A (2008). National innovation systems: A proposed framework for developing countries. International Journal of Entrepreneurship and Innovation Management.

[CR52] Keller W (2002). Trade and the transmission of technology. Journal of Economic Growth.

[CR53] Keller W (2004). International technology diffusion. Journal of Economic Literature.

[CR03] Kersan-Škabić I (2019). The drivers of global value chain (GVC) participation in EU member states. Economic research-Ekonomska istraživanja.

[CR55] Lee E, Yi K (2018). Global value chains and inequality with endogenous labor supply. Journal of International Economics.

[CR56] Lenzen M, Kanemoto K, Moran D, Geschke A (2012). Mapping the structure of the world economy. Environmental Science and Technology.

[CR57] Lenzen M, Moran D, Kanemoto K, Geschke A (2013). Building Eora: A global multi-regional input–output database at high country and sector resolution. Economic Systems Research.

[CR58] Malerba F, Mancusi ML, Montobbio F (2013). Innovation, international R&D spillovers and the sectoral heterogeneity of knowledge flows. Review of World Economy.

[CR60] Marshall G, Parra Á (2019). Innovation and competition: The role of the product market. International Journal of Industrial Organization.

[CR61] Maskell P, Malmberg A (1999). Localized learning and industrial competitiveness. Cambridge Journal for Economics.

[CR63] McCalman P (2005). Who enjoys TRIPS abroad? An empirical analysis of intellectual property rights in the Uruguay round. The Canadian Journal of Economics.

[CR64] Mudambi R (2008). Location, control and innovation in knowledge intensive industries. Journal of Economic Geography.

[CR65] Namazi M, Mohammadi E (2018). Natural resource dependence and economic growth: A TOPSIS/DEA analysis of innovation efficiency. Resources Policy.

[CR66] O’Donoghue T, Zweimuller L (2004). Patents in a model of endogenous growth. Journal of Economic Growth.

[CR67] OECD (2005). Guidelines for collecting and interpreting innovation data: Oslo manual.

[CR68] OECD (2013). Upgrading in global value chains: The role of knowledge-based capital in interconnected economies: Benefiting from Global Value Chains. OECD Publishing.

[CR69] OECD (2013). Supporting investment in knowledge capital, growth and innovation. OECD Publishing.

[CR70] OECD (2017). The links between global value chains and global innovation networks. OECD Publishing.

[CR71] Pietrobelli C (2008). Global value chains in the least developed countries of the world: Threats and opportunities for local producers. International Journal of Technological Learning, Innovation and Development.

[CR73] Pipkin S, Fuentes A (2017). Spurred to upgrade: A review of triggers and consequences of industrial upgrading in the global value chain. World Development.

[CR74] Raghupathi V, Raghupathi W (2019). Exploring science and technology led innovation: A cross country study. Journal of Innovation and Entrepreneurship.

[CR75] Rodrik, D. (2018). New technologies, global value chains, and developing economies. *Diffusion of Innovation eJournal*.***

[CR76] Schmidt M (1997). Managerial incentives and product market competition. Review of Economic Studies.

[CR77] Schmitz H (2004). Local enterprises in the global economy.

[CR78] Schmitz H, Knorringa P (2000). Learning from global buyers. Journal of Development Studies.

[CR79] Scotchmer S, Green J (1990). Novelty and disclosure in patent law. RAND Journal of Economics.

[CR80] Tajoli L, Felice G (2018). Global value chains participation and knowledge spillovers in developed and developing countries. The European Journal of Development Research.

[CR81] Tavassoli S (2018). The role of product innovation on export behavior of firms: Is it innovation input or innovation output that matters?. European Journal of Innovation Management.

[CR82] UNCTAD. (2022). Non-tariff measures from A to Z. https://unctad.org/system/files/official

[CR83] Vishwasrao S, Gupta S, Benchekroun S (2007). Optimum tariffs and patent length in a model of north-south technology transfer. International Review of Economics and Finance.

[CR84] Werner M (2012). Beyond upgrading: Gendered labor and the restructuring of firms in the Dominican Republic. Economic Geography.

[CR85] WIPO (2017). World Intellectual Property Report 2017: Intangible capital in global value chains.

[CR86] WIPO (2021). World intellectual property indicators 2021.

[CR87] World Bank (2020). World Development Report 2020. Trading for development in the age of global value chains.

[CR88] Youssef J, Zaki C (2022). A decade of competition laws in Arab economies: A dejure and de facto assessment. International Journal of Economic Policy in Emerging Economies.

[CR90] Zhang X, Wan G, Li J, He Z (2020). Global spatial economic interaction: Knowledge spillover or technical diffusion?. Spatial Economic Analysis.

