# Supplementary material for: On GVC and innovation: the moderating role of policy
Source: J. Ind. Bus. Econ. 2023 Jan 3;50(1):49–71. doi: 10.1007/s40812-022-00255-9 (PMC9808736; doi:10.1007/s40812-022-00255-9)
Supplement: Supplementary file 1 — Supplementary file1 (DOCX 38 KB) [file 40812_2022_255_MOESM1_ESM.docx]

**Appendix**

**Table A1** EORA26 sectors

| Agriculture | Construction |
| --- | --- |
| Fishing | Maintenance and Repair |
| Mining and Quarrying | Wholesale Trade |
| Food & Beverages | Retail Trade |
| Textiles and Wearing Apparel | Hotels and Restaurants |
| Wood and Paper | Transport |
| Petroleum, Chemical and Non-Metallic Mineral Products | Post and Telecommunications |
| Metal Products | Financial Intermediation and Business Activities |
| Electrical and Machinery | Public Administration |
| Transport Equipment | Education, Health and Other Services |
| Other Manufacturing | Private Households |
| Recycling | Others |
| Electricity, Gas and Water | Re-export & Re-import |

Source: EORA26 online dataset.

**Table A2** Dataset countries and income groups classification

|  | **Country** | **Income Group** |  | **Country** | **Income Group** |
| --- | --- | --- | --- | --- | --- |
| **1** | Algeria | Upper Middle | **43** | Lithuania | High |
| **2** | Argentina | Upper Middle | **44** | Luxembourg | High |
| **3** | Armenia | Upper Middle | **45** | Madagascar | Low |
| **4** | Australia | High | **46** | Malta | High |
| **5** | Austria | High | **47** | Malaysia | Upper Middle |
| **6** | Bangladesh | Lower Middle | **48** | Mexico | Upper Middle |
| **7** | Belarus | Upper Middle | **49** | Moldova | Lower Middle |
| **8** | Belgium | High | **50** | Monaco | High |
| **9** | Brazil | Upper Middle | **51** | Mongolia | Low |
| **10** | Bulgaria | Upper Middle | **52** | Morocco | Lower Middle |
| **11** | Canada | High | **53** | Netherlands | High |
| **12** | Chile | High | **54** | New Zealand | High |
| **13** | China | Upper Middle | **55** | Norway | High |
| **14** | Colombia | Upper Middle | **56** | Pakistan | Lower Middle |
| **15** | Costa Rica | Upper Middle | **57** | Peru | Upper Middle |
| **16** | Croatia | High | **58** | Philippines | Lower Middle |
| **17** | Cuba | Upper Middle | **59** | Poland | High |
| **18** | Czech Republic | High | **60** | Portugal | High |
| **19** | Denmark | High | **61** | Romania | Upper Middle |
| **20** | Ecuador | Upper Middle | **62** | Russian Federation | Upper Middle |
| **21** | Egypt | Lower Middle | **63** | Saudi Arabia | High |
| **22** | Finland | High | **64** | Singapore | High |
| **23** | France | High | **65** | Slovak Republic | High |
| **24** | Georgia | Upper Middle | **66** | South Africa | Upper Middle |
| **25** | Germany | High | **67** | Spain | High |
| **26** | Greece | High | **68** | Sri Lanka | Upper Middle |
| **27** | Guatemala | Upper Middle | **69** | Sweden | High |
| **28** | Hong Kong | High | **70** | Switzerland | High |
| **29** | Hungary | High | **71** | Syria | Low |
| **30** | Iceland | High | **72** | Tajikistan | Low |
| **31** | India | Lower Middle | **73** | Thailand | Upper Middle |
| **32** | Indonesia | Lower Middle | **74** | Tunisia | Lower Middle |
| **33** | Iran | High | **75** | Turkey | Upper Middle |
| **34** | Israel | High | **76** | Ukraine | Lower Middle |
| **35** | Italy | High | **77** | United Kingdom | High |
| **36** | Jamaica | Upper Middle | **78** | United States | High |
| **37** | Japan | High | **79** | Uruguay | High |
| **38** | Kazakhstan | Upper Middle | **80** | Uzbekistan | Lower Middle |
| **39** | Kenya | Lower Middle | **81** | Venezuela | Upper Middle |
| **40** | Korea | High | **82** | Vietnam | Lower Middle |
| **41** | Kyrgyz Republic | Lower Middle | **83** | Zambia | Lower Middle |
| **42** | Latvia | High |  | | |

Source: Authors’ own elaboration using the World Development Indicators online dataset.

**Table A3** Descriptive statistics

| Variable | Definition | Obs. | | Mean | Std. Dev. | Min | Max |
| --- | --- | --- | --- | --- | --- | --- | --- |
| Log (Resident patent per capita) | Number of resident patent applications for process, design, and products divided by the total population expressed in logarithm | | 2,490 | -4.6 | .927 | -7.313 | -2.479 |
| GVCRD | R&D weighted GVC | | 2,490 | .591 | .295 | .018 | 1 |
| Log (RD Stock) | Number of researches in R&D per million people expressed in logarithm | | 2,490 | 2.92 | .679 | .871 | 4.917 |
| Log (GDP per capita) | GDP per capita in constant 2010 US$ expressed in logarithm | | 2,490 | 3.945 | .607 | 2.565 | 5.321 |
| Log (Population) | Total number of people living in the country expressed in logarithm | | 2,490 | 7.224 | .732 | 4.469 | 9.145 |
| Tariffs | Weighted average of effectively applied rates on manufactured products | | 2,490 | 6.646 | 8.188 | 0 | 90.39 |
| Fuel Exports | Oil exports as a percentage of merchandise exports | | 2,490 | 13.87 | 21.94 | 0 | 98.764 |
| Log (Time to Contracts) | Number of days to enforce contracts expressed in logarithm | | 2,490 | 2.721 | .214 | 2.079 | 3.233 |
| Rule of Law | Percentile rank among all countries | | 2,490 | 58.653 | 28.706 | .469 | 100 |
| TRIPS | Signing of a deep trade agreement involving intellectual property rights | | 2,490 | .556 | .497 | 0 | 1 |
| WIPO | Being a WIPO member | | 2,490 | .734 | .442 | 0 | 1 |
| NTMs | Comprehensive trade costs with main trading partner excluding tariffs indicator | | 2,050 | 75.368 | 34.689 | -1.98 | 188.423 |
| Anti-Monopoly Index | The effectiveness of the anti-monopoly law index | | 2,075 | 4.232 | .912 | 2.31 | 6.192 |
| Log (non-resident patent) | Number of non-residents patent applications for process, design, and products divided by the total population expressed in logarithm | | 2,490 | -10.326 | 1.915 | -16.048 | -5.694 |
| RD expenditures | R&D expenditures as a percentage of GDP | | 2,430 | .908 | .905 | .005 | 5.14 |
| GVC | Share of exported foreign value added from TiVA dataset | | 1,368 | 24.71 | 11.55 | 2.52 | 68.16 |
| IMRD | R&D weighted imports | | 2,460 | 1.445 | 1.071 | .0173 | 8.169 |

Source: Authors’ own elaboration.

**Table A4** Effect of alternative IPRs agreements on resident patent per capita

|  | Dependent Variable: Log of Resident Patent per capita | | | |
| --- | --- | --- | --- | --- |
|  | (1) | (2) | (3) | (4) |
| GVCRD | 2.812*** | 2.879*** | 2.901*** | 2.655*** |
|  | (.326) | (.325) | (.326) | (.335) |
| Log (RD Stock) | .94*** | .945*** | .949*** | .844*** |
|  | (.105) | (.104) | (.104) | (.11) |
| Log (GDP per capita) | .42*** | .395*** | .389*** | .369*** |
|  | (.088) | (.089) | (.089) | (.089) |
| Log (Population) | .73*** | .585*** | .583*** | .552*** |
|  | (.173) | (.177) | (.177) | (.177) |
| Tariffs | -.012*** | -.011*** | -.011*** | -.011*** |
|  | (.001) | (.001) | (.001) | (.001) |
| Fuel Exports | -.003*** | -.003*** | -.003*** | -.003*** |
|  | (.001) | (.001) | (.001) | (.001) |
| Log (Time to Contracts) | -.051 | -.026 | -.022 | -.026 |
|  | (.142) | (.142) | (.142) | (.141) |
| Rule of Law | .004*** | .004*** | .004*** | .004*** |
|  | (.001) | (.001) | (.001) | (.001) |
| RD*GVCRD | -.886*** | -.907*** | -.917*** | -.797*** |
|  | (.107) | (.106) | (.107) | (.113) |
| WIPO |  | .081*** | -.02 | .043 |
|  |  | (.023) | (.097) | (.099) |
| WIPO*low-excluded |  |  | .106 | .195* |
|  |  |  | (.099) | (.102) |
| WIPO*GVCRD |  |  |  | -.22*** |
|  |  |  |  | (.071) |
| Constant | -14.45*** | -13.422*** | -13.396*** | -12.853*** |
|  | (1.531) | (1.554) | (1.555) | (1.562) |
| No. of Observations  No. of Countries | 2,490  83 | 2,490  83 | 2,490  83 | 2,490  83 |
| R^2^ | .178 | .182 | .182 | .186 |
| Country FE | Yes | Yes | Yes | Yes |
| Year FE | Yes | Yes | Yes | Yes |
| *Notes:* Standard errors are in parentheses. Fixed effects are removed for brevity. *** p<.01, ** p<.05, * p<.1 WIPO is a dummy variable equals 1 if the country is a WIPO member and equals zero otherwise. | | | | |
|  | | | | |
